# Static and dynamic properties of decane/water microemulsions stabilized by cetylpyridinium chloride cationic surfactant and octanol cosurfactant

**DOI:** 10.1039/d0ra06313d

**Published:** 2020-10-01

**Authors:** M. Lemaalem, R. Ahfir, A. Derouiche, M. Filali

**Affiliations:** Laboratoire de Physique des Polymères et Phénomènes Critiques Sciences Faculty Ben M’Sik, Hassan II University P. O. Box 7955 Casablanca Morocco mohammedlemaalem@gmail.com; Laboratory of Theoretical and Applied Physics (LPTA), Sidi Mohamed Ben Abdellah University, Faculty of Sciences Dhar El Mahraz BP 1796 Fes, Atlas Morocco

## Abstract

Molecular dynamics simulation (MD) is used to study the static and dynamic properties of positively charged decane/water microemulsions, for various volume fractions *Φ* (2.8%, 6.98%, 14%, and 26.5%). An effective hybrid potential combining three potentials, namely the hard-sphere repulsive potential, the van der Waals attractive potential, and the Yukawa repulsive potential, is used to describe the microemulsion interactions. The microemulsion shape is determined using the renormalized spectra in Porod representation. The appropriate potential parameters are tested using the Ornstein–Zernike integral equation approach with the Hypernetted Chain (HNC) closure relation by a comparison between the structure factor calculated from HNC and that obtained from Small Angle Neutron Scattering experiments (SANS). Thus, the micro arrangements of microemulsions have been analyzed using the pair correlation function *g*(*r*) and the structure factor *S*(*q*) obtained from HNC, SANS, and MD simulation using these parameters. The microemulsion dynamic properties were discussed using the mean-square displacement (MSD) and the diffusion coefficient *D*_c_ calculated from MD simulations.

## Abstract 2

We report a molecular dynamics simulation using a proven effective interaction potential to study the physical properties of decane/water microemulsions.

## Graphical abstract

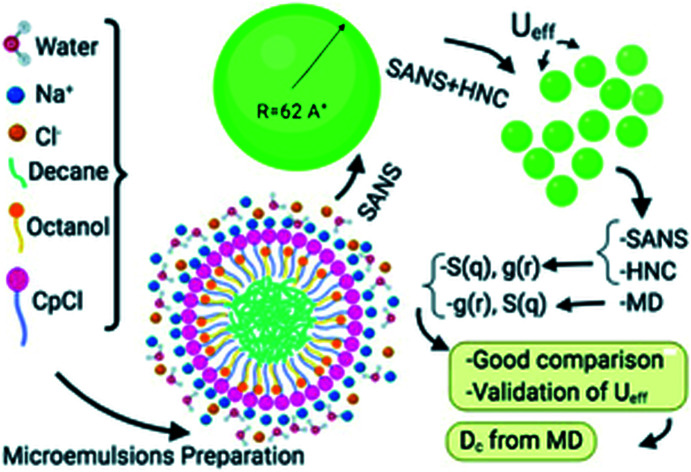

## 1 Introduction

Microemulsions possess interesting physicochemical properties, thermodynamic stability, low interfacial tension, low viscosity, transparency, and high solubilization power.^1–3^ From an industrial viewpoint, microemulsions have a simple and low-cost preparation process. The properties of microemulsions make them useful for transdermal drug delivery;^4,5^ they can provide rapid drug release due to the large interfacial area and this improves the efficacy of the drug. Also, they can solubilize both hydrophilic and lipophilic drugs. Usually, microemulsions comprise two immiscible liquids and a mixture of surfactant and cosurfactant. High amounts of surfactant and cosurfactant provide an enormous reduction in interfacial tension,^6^ enabling a large interface and supporting the spontaneous formation of microemulsions. The low interfacial tension ensures excellent surface contact between the skin and the microemulsion and facilitates further skin penetration. Surfactants decrease the interfacial tension, thus facilitating dispersion during the preparation of microemulsions. They form a flexible film which provides the correct curvature for the interfacial region. Hydrophilic surfactants were found to increase percutaneous absorption whereas less hydrophilic surfactants increased skin drug deposition.^7^ The cosurfactant provides the flexibility required by the interfacial film to form microemulsions with different curvatures. Short to medium alcohol chains are used as cosurfactants to decrease the interfacial tension and increase fluidity.^8–10^ Thus, microemulsions may optimize skin drug targeting and reduce further systemic absorption.

From a mesoscopic point of view, much effort has been dedicated to the development and parameterization of effective interaction potentials to study the structure and dynamics of colloidal systems.^11–13^ This is supposed to save extensive details and yield valuable information. Thus, the physical properties, in terms of the micro arrangements and collective dynamics of colloidal systems, are determined by considering the colloids as dispersed particles interacting *via* an effective interaction. In this context, a series of reports have studied the theoretical modeling and numerical simulation of microemulsions, aiming, in particular, to determine their structure and dynamic properties using MD simulations,^12,13^ using an effective interaction of the Sogami–Ise type. This work could make a substantial contribution to determining the dynamic laws of Pickering emulsions. However, it has not yet been confirmed by enough experimental results. In the present study, we have modeled the interactions between positively charged microemulsions using hard sphere, van der Waals, and electrostatic repulsion potentials.

**Fig. 1:**
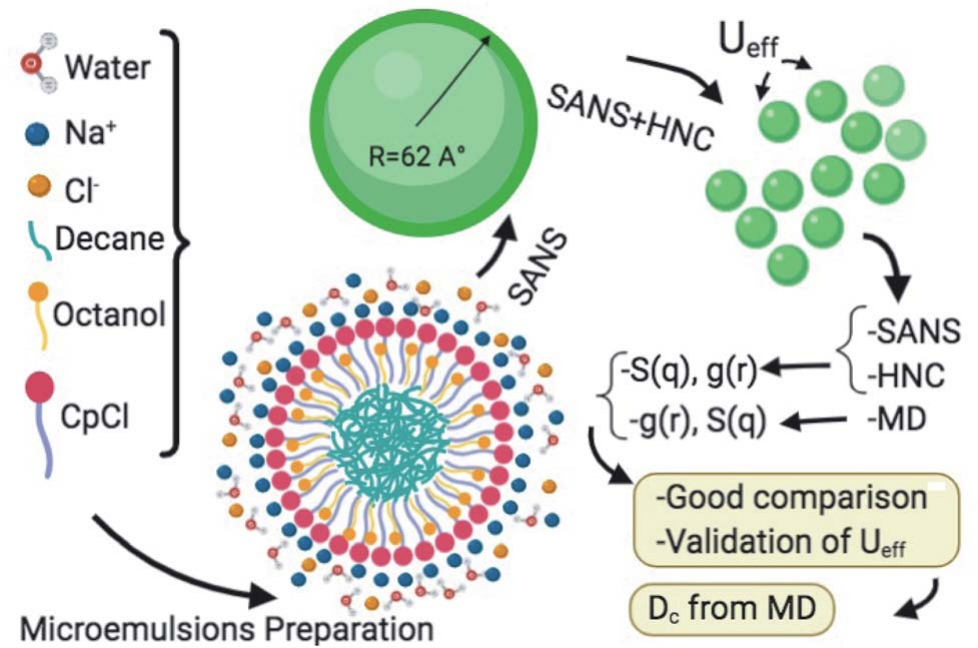
Schematic representation of the research design and perspectives.

Fig. 1 shows a schematic representation of the main research steps. Accordingly, the proposed interaction potential model is tested by a numerical solution of the Ornstein–Zernike (OZ) integral equation with the Hypernetted Chain (HNC) approximation.^14–18^ Then, we verify the validity of the potential parameters with molecular dynamics simulation and compare the results of MD, HNC approximation, and SANS experiments for the structure factor *S*(*q*). Furthermore, MD is used to determine the thermodynamical properties of the system, a microemulsion of decane in salt-water stabilized by a monolayer of cetylpyridinium chloride ionic surfactant and octanol cosurfactant.

## 2 Materials and methods

### 2.1 Materials

Cetylpyridinium chloride [H_3_C–(CH_2_)_15_]–C_5_H_5_N^+^Cl^−^ (CpCl) was purchased from Fluca and was purified by successive recrystallization in water and in acetone. Octanol [H_3_C–(CH_2_)_7_]–OH and decane [H_3_C–(CH_2_)_8_CH_3_] were obtained from Fluca and were used as received. All samples were prepared by weight in brine or deuterated brine. Brine (0.2 M NaCl) was prepared with triply distilled water or deuterated water according to the Material Safety Data Sheet (MSDS).

### 2.2 Microemulsion preparation

The surfactant film was composed of a CpCl cationic surfactant and an octanol cosurfactant. The weight ratio of octanol to CpCl was 0.25 and the weight ratio of decane to the surfactant film was 0.62. The samples were shaken and kept in a water bath for ten days at a constant temperature. After this time, phase separation was clearly observed. Then, the samples were homogenized, left to rest, and observed for four days to ensure their thermodynamic stability.

The weight ratio of octanol to CpCl is chosen to be just below the emulsification failure limit, which refers to the limit above which the microemulsion droplets are saturated with oil and coexist with excess oil. Under such conditions, the microemulsion droplets are spheres with a well-defined radius.^19–21^ The prepared microemulsions can be diluted over a large concentration range (1–20 wt%). In order to properly study the structural and dynamic properties at different microemulsion concentrations, we performed the study on more than ten samples (1.4%, 2.8%, …, 26%). After analysis of these samples, we noticed that the results for the volume fractions 2.8%, 6.98%, 14%, and 25.5% describe the behavior of our system well.

### 2.3 Small angle neutron scattering (SANS) measurements

The SANS experiments were performed at LLB-Saclay on the PACE spectrometer. The configurations used were 1.5 m at 6 Å and 4.68 m at 13 Å. The range of scattering vectors covered was 0.004 Å^−1^ < *q* < 0.16 Å^−1^ and the temperature was *T* = 293 K. The scattering data are treated according to standard procedures. They are put on an absolute scale by using water as a standard. And the obtained intensities in absolute units (cm^−1^) are found with accuracy better than 10%. All spectra models are convoluted by the instrument response function, taking into account the uncertainty of the neutron wavelength and the angular definition.^22^ In order to separate the form factor *P*(*q*) from the structure factor *S*(*q*), it is necessary to measure the scattering intensity *I*(*q*). For a finite microemulsion concentration, the scattering due to one particle *P*(*q*) is isolated. Thus, the form factor, *P*(*q*), is found. Then the scattering intensity is measured in order to find the structure factor, *S*(*q*). The size of the microemulsion is determined using SANS experiments with sphere contrast. For all studied samples, the scattering is essentially due to the microemulsions. Thus, the scattered intensity *I*(*q*) is modeled using the following expression:^23^1
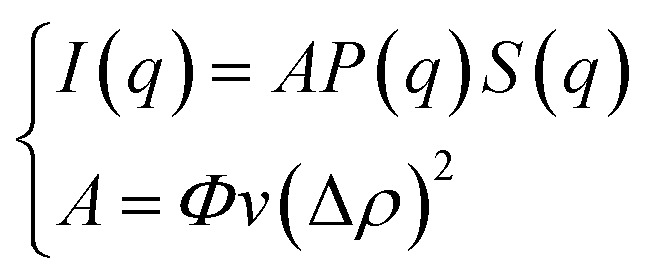
where *S*(*q*) is the structure factor, *q* (Å^−1^) is the scattering vector, *Φ* is the volume fraction of the aggregates, *v* (cm^3^) is the dry volume of the aggregates, Δ*ρ* = 6.83 × 10^10^ cm^−2^ is the contrast, *i.e.*, the difference between the aggregate and the solvent scattering length density, and *P*(*q*) is the form factor of the colloidal aggregates, such that *P*(*q* → 0) = 1.

### 2.4 Structure and thermodynamics from the integral equation method

The physical properties of microemulsions are essentially determined by the interactions between particles which are in most cases modeled by a pair potential. However, other contributions should be taken into account. The central problem in liquid theory is the determination of the radial distribution function *g*(*r*) of the system particles. The radial distribution function is important for several reasons; its Fourier transform is the structure factor *S*(*q*), which can be measured experimentally by neutron scattering, thus allowing a direct comparison between theory and experiment. The *g*(*r*) is related to the mean interaction potential by:2
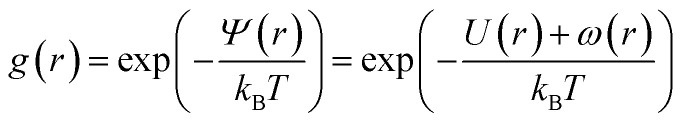
The average effective potential *Ψ*(*r*) includes not only the effect of direct interactions *U*(*r*) between a particle located at the origin and other surrounding particles but also the average effect of the forces of the neighboring particles which surround each particle *ω*(*r*). The structure factor *S*(*q*) is the Fourier transform of the radial distribution function *g*(*r*), which describes the three-dimensional distribution arrangement of microemulsions, where3
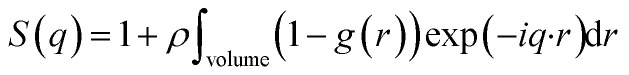
The isothermal compressibility *χ*_T_ is related to the structure factor *S*(*q*) at *q* → 0:

4 *ρk*_B_·*χ*_T_ = *S*(0) = 1 + 4π*ρ*∫(1 − *g*(*r*))*r*^2^d*r*

The thermodynamic quantities of the microemulsion depend on *g*(*r*) and *U*(*r*). We write the first relationship giving the internal energy as a summation of the kinetic and potential energies:5
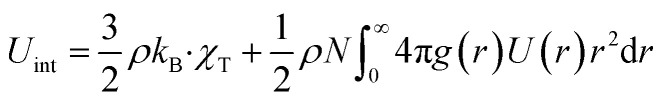
Also, the system pressure can be expressed as a function of *g*(*r*), by connecting the 2nd virial coefficient to *g*(*r*):6
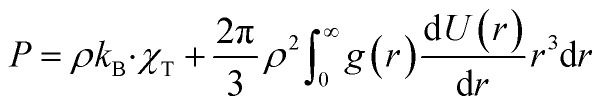


### 2.5 Solution of the Ornstein–Zernike integral equation

The second step consists of determining the RDF by solving the well-known Ornstein–Zernike (OZ) integral equation. The latter is solved by the total-correlation-function, *h*(*r*) = *g*(*r*) − 1,^24^

7 *h*(*r*) = *ρ*∫*c*(|*r* − *r*′|)*h*(*r*′)d*r*′

Here *c*(*r*) represents the direct-correlation-function. Since the considered system is homogeneous and isotropic, the associated functions *g*(*r*), *h*(*r*) and *c*(*r*) depend only on the particle distance, *r*, where one particle is taken as the origin. Then, *r* is the position-vector of the second particle.

Fourier transforming the above integral equation and taking into account relation (3) yields the following general formal expression for *S*(*q*).8
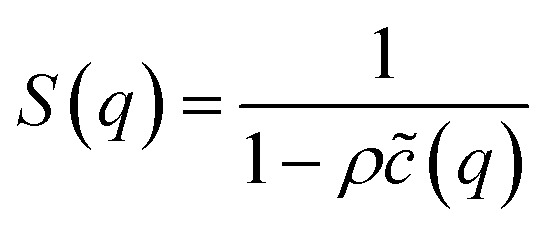
where *c̃*(*q*) is the Fourier transform of the direct-correlation-function, *c*(*r*). The OZ equation (eqn (7)) contains two unknown functions which are *c*(*r*) and *h*(*r*). Therefore, to get *h*(*r*) (and thus *g*(*r*)), we need an additional equation (closure relation) connecting *h*(*r*) and *c*(*r*) to the pair potential, *U*(*r*). Within the framework of HMSA, the adopted closure relation is as follows^24^

9 *c*^HMSA^ = exp{−*βU*(*r*) + *Γ*(*r*) + *B*^HMSA^(*r*)} − 1 − *Γ*(*r*)

with the associated bridge-function10
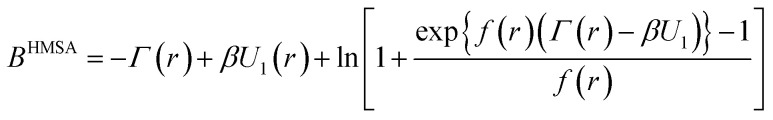
Generally, such a function is defined as an infinite series of correlation diagrams. We note that HMSA interpolates between “Soft Core Mean Spherical” and HNC approximations. In eqn (9) and (10), the function *Γ*(*r*) is the difference between the total and direct-correlation-functions, *i.e.*, *Γ*(*r*) = *h*(*r*) − *g*(*r*), and the interaction potential, *U*(*r*), is divided into a short-range part, *U*_0_(*r*), and a long-range attractive tail, *U*_1_(*r*), as prescribed by Weeks *et al.*^25^ We have

11 *U*(*r*) = *U*_0_(*r*) + *U*_1_(*r*)

with12
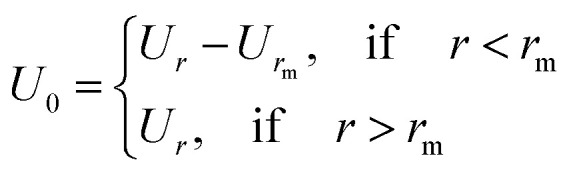
and13
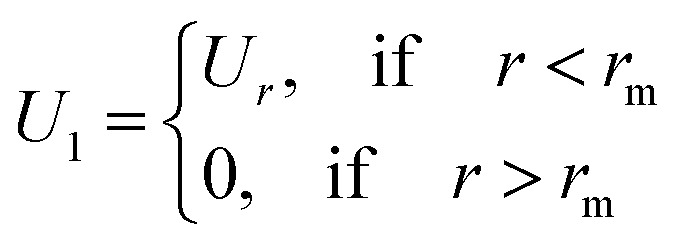
Here *r*_m_ denotes the position of the minimum of the overall potential, *U*(*r*). The above decomposition was proposed by Weeks, Chandler, and Anderson.^25^ In eqn (10), the quantity *f*(*r*) represents the mixing-function.^26^ A new form of this function was suggested by Bretonnet and Jakse.^27^ Its advantage is that it ensures thermodynamic consistency in the calculation of the isothermal compressibility by two different ways. These authors have proposed the following form for the mixing-function

14 *f*(*r*) = *f*(0) + (1 − *f*(0))exp(−*ar*)

where *a*^−1^ is some characteristic length and *f*(0) is the interpolation constant. The latter is an adjustable parameter such that 0 ≤ *f*_0_ ≤ 1. This constant that serves to eliminate thermodynamic inconsistency can be fixed by equating the compressibility deduced from the virial pressure, using eqn (6), to that calculated from the zero-scattering-angle limit of the structure factor using eqn (4).

### 2.6 Molecular dynamics simulation (MD)

#### 2.6.1 Interaction potential

Microemulsions are approximated as non-overlapping hyperspherical uniformly charged particles, interacting by an effective potential based on the DLVO model,^28,29^ which is the sum of a hard sphere repulsive potential, a van der Waals attractive term^28^ and a Yukawa type screened coulombic repulsive potential:^29^

**Fig. 2:**
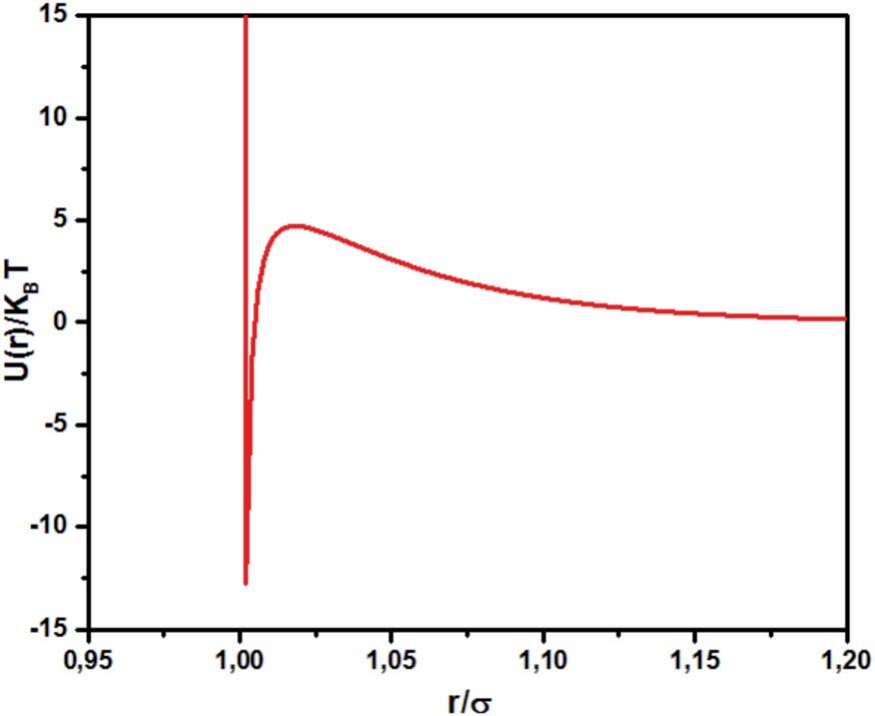
Dimensionless pair potential, *U*(*r*)/*k*_B_*T*, *versus* the dimensionless distance *r*/*σ* (*σ* = 2*R*).

15 *U*(*r*) = *U*_HS_ + *U*_VW_ + *U*_Coul_

with:16
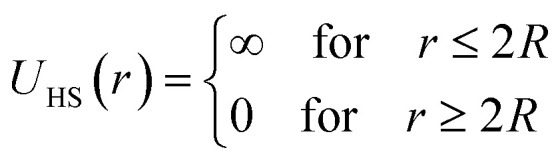
17
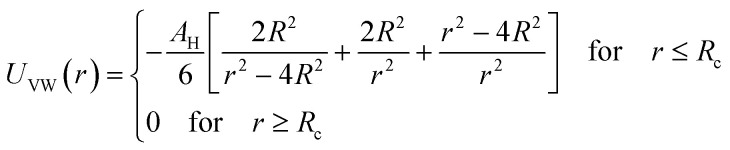
where *r* is the center-to-center distance and *R* is the microemulsion radius. *A*_H_ is the Hamaker constant, with *A*_H_ = 1.1*k*_B_*T*; this value is appropriate for decane microemulsions interacting through water.^30^*R*_c_ is the cut off distance for the interaction potential, *R*_c_ = 12*R*.18
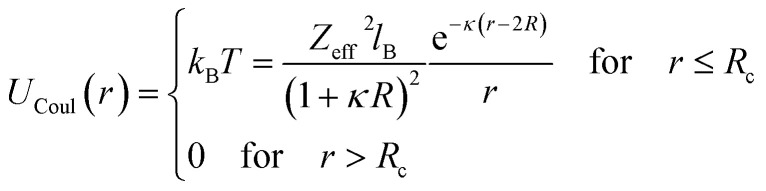
where *l*_B_ is the Bjerrum length, *l*_B_ = *e*^2^/4*κ*^−1^*ε*_0_*ε*_r_*k*_B_*T* = 7.12 Å, where *ε*_r_ = 80 is the dielectric constant of water. *T* is the temperature, and *κ*^−1^ represents the Debye length, *κ*^−1^ = 6.7 Å, which corresponds to the concentration of small ions added to the solution. *Z*_eff_ is the number of effective charges per microemulsion, *Z*_eff_ = 130 for each microemulsion. Fig. 2 presents the interaction potential plotted using these parameters.

#### 2.6.2 Simulation details

**Fig. 3:**
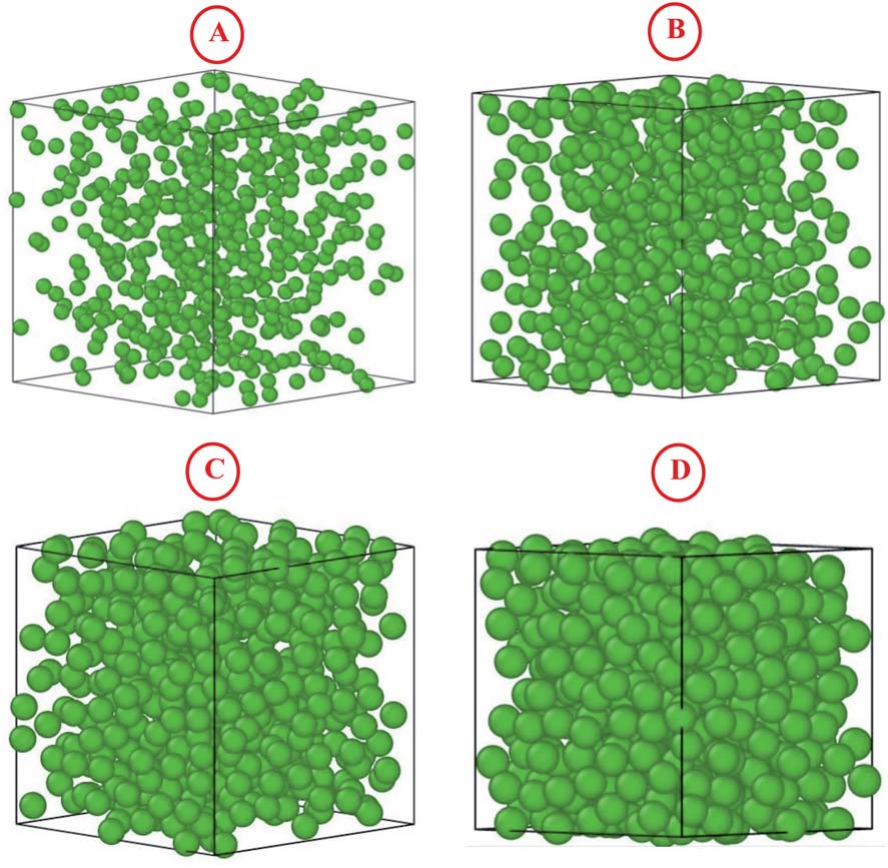
The simulated microemulsions for the four studied volume fractions *Φ*: (A) *Φ* = 2.8%, (B) *Φ* = 6.98%, (C) *Φ* = 14%, and (D) *Φ* = 26.5%.

Molecular dynamic simulations are performed in the NVT statistical ensemble, where *N* = 10^6^ is the number of simulated microemulsions, *V* is the volume of the simulation box and *T* is the temperature. Periodic boundary conditions in three dimensions are applied to remove the surface effect.^31^ Dimensionless units are used to optimize the calculation time.^31,32^ Accordingly, the distance is reduced by the microemulsion radius *R*, the energies are reduced by *k*_B_*T*, the time is reduced by 
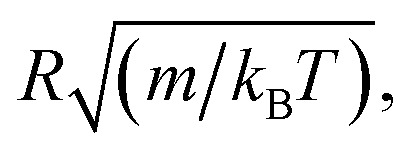
 the pressure *P* is reduced by *k*_B_*T*/*R*^3^, the isothermal compressibility *χ*_T_ is reduced by *R*^3^/*k*_B_*T*, the diffusion coefficient *D*_c_ is reduced by 
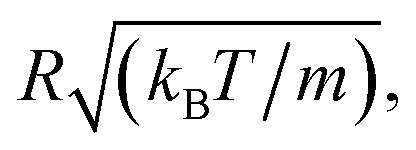
 the viscosity *η* is reduced by 
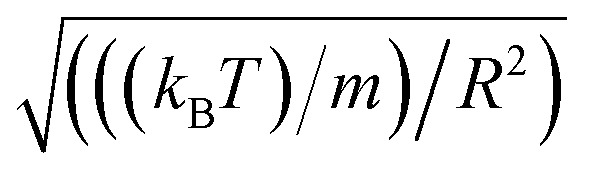
 and the dimensionless temperature is set to T* = 1. The equations of motion are integrated using the Verlet algorithm^33^ for one million steps with a time step δ*t* = 0.001. All molecular dynamics simulations were performed using the LAMMPS simulation package (Fig. 3).^34^

## 3 Results and discussions

### 3.1 Microemulsion shape

**Fig. 4:**
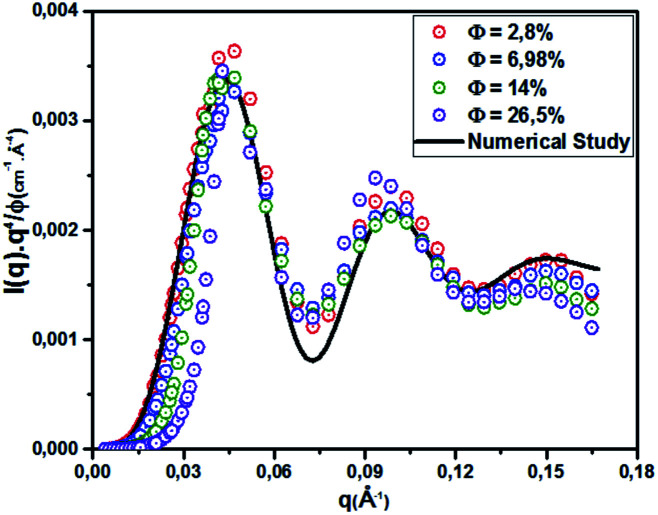
Renormalized spectra in Porod representation (*q*^4^·*I*(*q*)) at different volume fractions (*Φ* = 2.8%, *Φ* = 6.98%, *Φ* = 14%, and *Φ* = 26.5%). The solid line is the computed spectrum for *R̄* = 62 Å and Δ*R* = 6.2 Å.

In all studied cases, the polydispersity of the microemulsion size is described by a Gaussian distribution with mean radius *R̄*, and standard deviation Δ*R*, assuming that microemulsions are spherical particles. Then, the average form factor of the polydisperse size of the studied microemulsions is:19
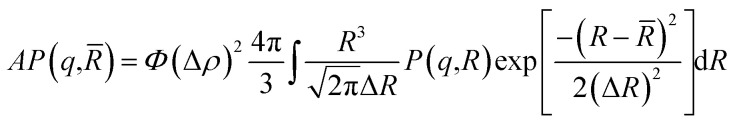
with the form factor20
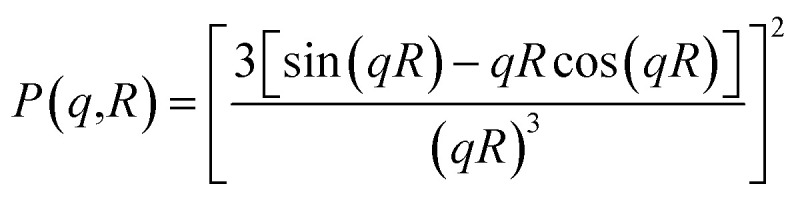
Here, *R* is the microemulsion radius. The Porod representation (*q*^4^·*I*(*q*)) as a function of *q* is used in order to amplify the *P*(*q*) oscillations, in the limit of large *q*.^35,37^ According to eqn (20) and Fig. 4, for the first and second maxima we find *qR* = 2.73 and *qR* = 6.12, and for the first and second minima, we find *qR* = 0 and *qR* = 4.99, respectively. Thus, for weakly polydisperse microemulsions, *R̄* has been estimated using eqn (19).

The re-normalized spectra are examined in the Porod representation (*q*^4^·*I*(*q*)/*Φ*) for different volume fractions ranging from *Φ* = 2.8% to *Φ* = 26.5%. All spectra display well-defined oscillations at intermediate and large *q* for all examined cases. These spectra are superimposed in the large *q* range, which indicates that the size and shape of the microemulsion remain constant for various *Φ*. This behavior is observed in many experimental reports investigating the microemulsion structure using the Porod representation.^35–37^ Assuming that decane/water microemulsions are spherical shaped, their radius is deduced from the maximum and minimum positions of the Porod representation. To investigate the size polydispersity, we assume a Gaussian distribution of the microemulsion radius. The spectra calculated using eqn (19) and (20) give a reasonable fit with mean radius *R̄* = 62 Å and polydispersity *p* = Δ*R*/*R̄* = 10%. For simplicity, the microemulsions are considered as monodisperse spheres of mean radius *R̄*.^38^

### 3.2 Static properties

**Fig. 5:**
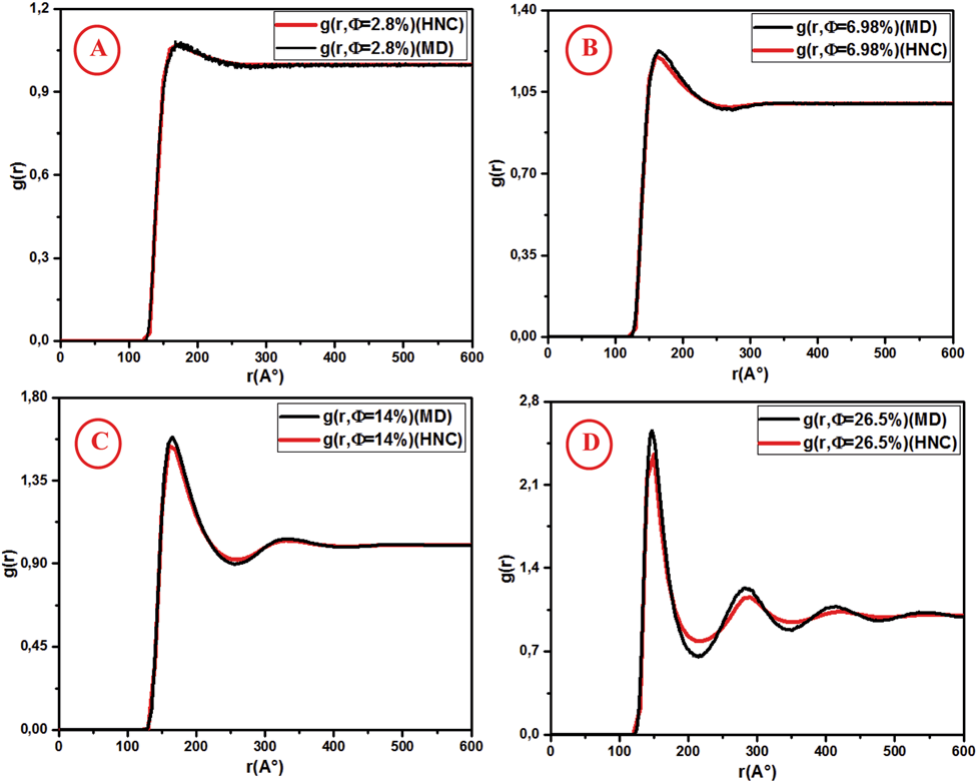
Radial distribution function *g*(*r*) from HNC and MD simulation, for different volume fractions: (A) *Φ* = 2.8%, (B) *Φ* = 6.98%, (C) *Φ* = 14% and (D) *Φ* = 26.5%.

Fig. 5 shows the pair correlation function *g*(*r*) as a function of the dimensionless distance *r*/*σ* obtained from the OZ integral equation with the HNC closure relation and MD simulation. The *g*(*r*) is calculated at various volume fractions (*Φ* = 2.8%, 6.98%, 14%, 26.5%). For the dilute systems (*Φ* = 2.8%, 6.98%), the depicted *g*(*r*) curves obtained from HNC and MD are in good agreement (Fig. 5A and B).

**Fig. 6:**
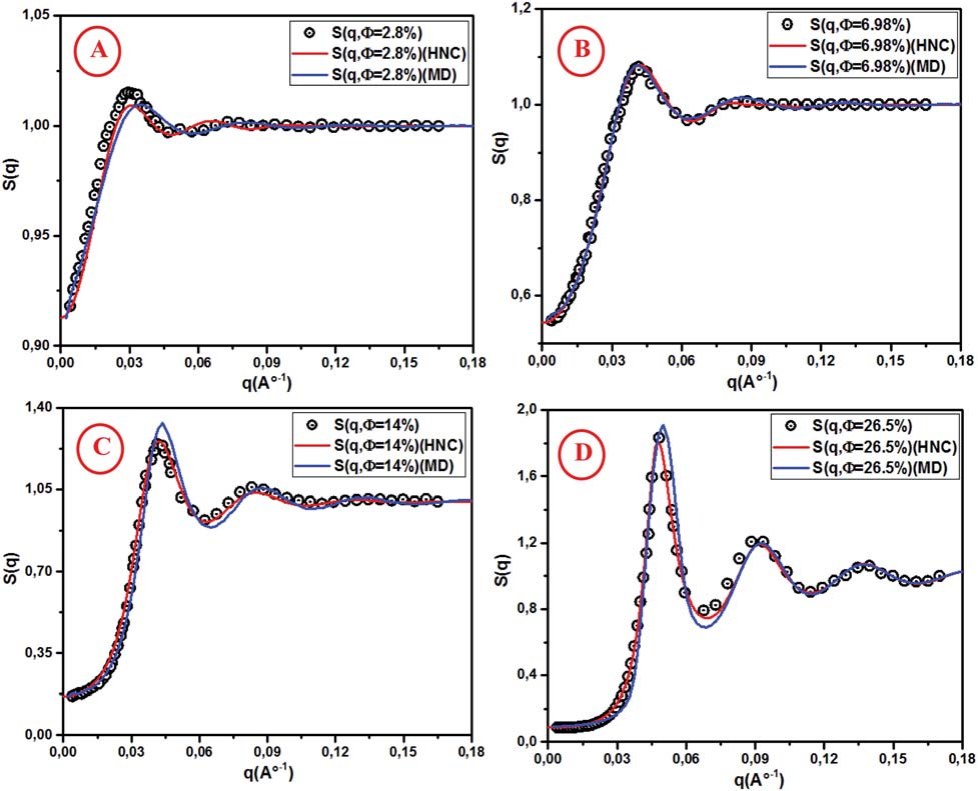
Structure factor *S*(*q*) from SANS, HNC and MD simulation, for different volume fractions: (A) *Φ* = 2.8%, (B) *Φ* = 6.98%, (C) *Φ* = 14% and (D) *Φ* = 26.5%.

For high concentrations (Fig. 5C and D), the HNC approximation slightly underestimates the principal peak height, *g*(*r*_m_), at the position *r*_m_, and the discrepancy between HNC and MD is noticeable for *Φ* = 26.5%. The microemulsion correlation increases on increasing the volume fraction *Φ* from 2.8% to 26.5%, and the height of the first *g*(*r*) peak increases from 1.07 to 2.36. Furthermore, the position of the principal peak is shifted to smaller values. This indicates that the interactions between microemulsions become more repulsive and the organization of the suspension structure is more ordered. Thus, the separation distance between microemulsions is shifted to smaller values, as can be observed in Fig. 5. Fig. 6 presents a comparison of the structure factors *S*_SANS_(*q*), *S*_HNC_(*q*), and *S*_MD_(*q*) obtained from the SANS experiment, HNC approximation, and molecular dynamics simulation, respectively. An excellent agreement is found for the studied spectra, proving that the proposed potential model is satisfactory for all studied volume fractions. The slight increase in the first peak height for *Φ* = 14% and *Φ* = 26.5% does not affect the fit quality as long as molecular dynamics simulation reproduces the peaks with the same *q*_max_. We notice that the principal peak becomes narrower and more pronounced with increasing *Φ*. Indeed, going from 2.8% to 26.5%, *S*_max_ varies from 1.012 to 1.833. On the other hand, the position of the main peak is shifted to higher values when *Φ* increases. This fact is not surprising because *q* is inversely proportional to the mean distance between microemulsions *d* (see Table 1). The present findings in terms of *g*(*r*) and *S*(*q*) are in good agreement with the results of other experimental and numerical approaches for similar colloidal systems.^11–13,39–41^ The results presented in Table 2 indicate that the pressure and the potential energy increase monotonically with increasing *Φ*, and the osmotic compressibility decreases with increasing *Φ*.

**Table 1:** Analysis of *S*(*q*) curves obtained by SANS, HNC and MD

Method	*Φ*	*S*(0)	*S*_max_	*d* (Å)
SANS	2.8%	0.9182	1.012	205
HNC		0.9185	1.009	204
MD		0.9180	1.0097	207
SANS	6.98%	0.548	1.087	153
HNC		0.544	1.083	152
MD		0.55	1.08	160
SANS	14%	0.164	1.247	148.4
HNC		0.167	1.247	148
MD		0.16	1.32	146
SANS	26.5%	0.088	1.833	130.6
HNC		0.087	1.850	130
MD		0.090	1.91	127

**Table 2:** Dimensionless static properties for different microemulsion volume fractions

*Φ*	Total energy/*Nk*_B_*T*	Pressure*	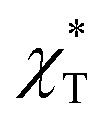
2.8%	1.510	0.007	137.015
6.98%	1.635	0.027	32.885
14%	1.903	0.109	4.996
26.5%	3.282	0.687	1.391

### 3.3 Dynamic properties

#### 3.3.1 Theoretical prediction of the microemulsion dynamic properties for low volume fractions

For low volume fractions, microemulsions experience Brownian dynamics. Thus, the Brownian motion of a microemulsion is described by the following phenomenological Langevin equation:21
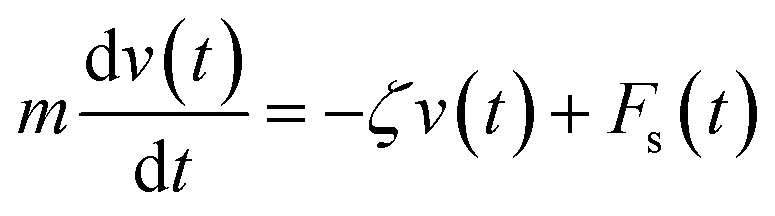
where *m* stands for the mass of the microemulsion, *v*(*t*) stands for its velocity, *ζ* stands for the friction coefficient, and *F*_s_(*t*) represents a random force. The friction coefficient *ζ* is related to the microemulsion viscosity, *η*, and the microemulsion radius, *R*, by the classical Stokes relation *ζ* = 6π*ηR*. The viscosity of the solution depends on the number density of the dispersed microemulsions 
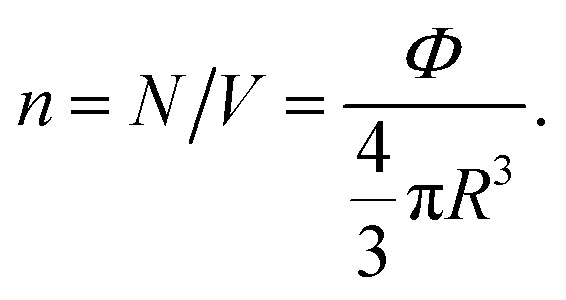
 For low volume fractions, the effective viscosity obeys the well-known law^42^22
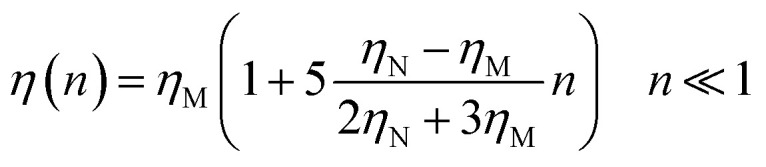
Here *n* accounts for the reduced microemulsion number density, *η*_M_ is the solvent viscosity, and *η*_N_ is the viscosity of the microemulsions. We have *η*(*n*) > *η*_M_, since *η*_N_ > *η*_M_. The stochastic force is considered to be white noise with:

23 〈*F*(*t*)〉 = 0

24 〈*F*_s_(*t*)·*F*_s_(0)〉 = 6*k*_B_*Tζδ*(*t*)

where *δ*(*t*) is the Dirac distribution. The brackets 〈…〉 mean an average over time. The mean-square displacement (MSD) 〈(*r* − *r*_0_)^2^〉 ≡ 〈*Δ*^2^*r*〉 is expressed as:25
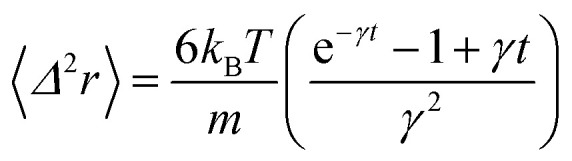
where *γ* = *ζ*/*m* is known as the relaxation rate. For times much longer than the inverse relaxation rate, that is for *t* ≫ *γ*^−1^ ≡ *τ*, the MSD increases linearly with time, and it is dependant on the diffusion coefficient *D*_c_, defined as the compromise between the thermal fluctuations and the dissipation such that 
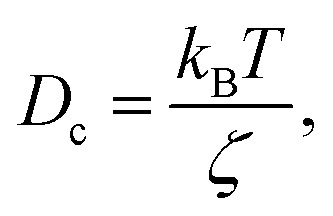
 according to:

26 〈*Δ*^2^*r*〉 = 6*D*_c_*t*, *t* ≫ *τ*

For *t* ≪ *τ*, the Taylor expansion of e^−*γt*^ gives: 
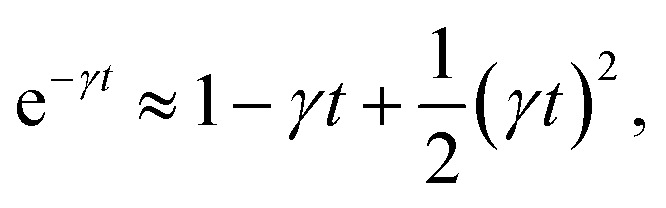
 and then we find:27
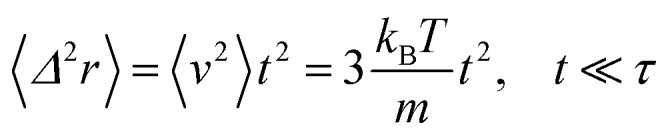


#### 3.3.2 Theoretical prediction of the microemulsion dynamic properties for high volume fractions

When the number density of the microemulsions reaches a critical density *n*_c_, their dynamics are more complex than those at low number density. We recall that, for low density, particles move ballistically for short times. Thus the mean-square displacement 〈*Δ*^2^*r*〉 ∼ *t*^2^, which is followed by a crossover to Fickian diffusion, characterized by 〈*Δ*^2^*r*〉 ∼ *t* for long times. But, in dense colloidal systems, a caging effect, where the particles are trapped by their neighbors, causes a subdiffusion regime intermediate between the ballistic and the diffusive regimes. This regime is characterized by 〈*Δ*^2^*r*〉 ∼ *t*^*α*^, with 0 ≤ *α* ≤ 1. For the time being, the theoretical prediction of the value of *α* in the presence of the cage effect remains poorly understood. But, in fact there are some theoretical approaches based on some complex memory functions developed to discuss the subdiffusion laws. From the literature, Zwanzig, in a series of studies, developed a theoretical approach based on a generalized Langevin equation (GLE),^42,43^ and this approach can be adopted to discuss the subdiffusion observed in microemulsions which can be considered as three dimensional colloidal particles swimming in a solvent. From a mathematical viewpoint this is simply an extension of the standard Langevin equation for simple liquids, where the friction is assumed to be determined by the instantaneous velocity of particles. It is noted that the difficulty of understanding the subdiffusion phenomenon lies in mathematically representing the cage effect that, physically speaking, depends on various physical parameters, namely, the temperature, the number density and the nature of the solvent. In this context, the GLE is expressed as28
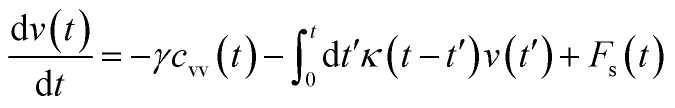
where *v* is the velocity of a moving microemulsion, 
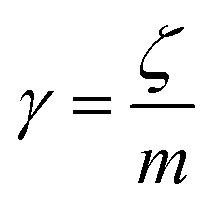
 is the relaxation rate, where *ζ* is the friction coefficient and *m* is the mass of the tracer, *κ* is the memory function that expresses the retardation due to friction, and *F*_s_(*t*) is a random force felt by the moving microemulsion due to its collisions with the solvent molecules. Thus, the random force is obtained from the equation

29 〈*F*_s_(*t*)·*F*_s_(0)〉 = 6*mk*_B_*T*[*γδ*(*t*) + *κ*(*t*)], *t* > 0

For these considerations, the VACF solves the following differential equation:30
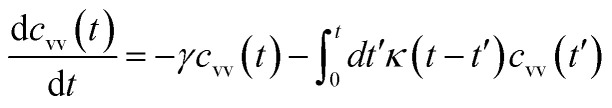
To solve the GLE, we adopt a memory function, recently proposed by Flenner, developed to study the dynamic aspect of lipid atoms. Flenner’s approach is based on the Zwanzig–Mori projection method for modeling 〈*Δ*^2^*r*〉. The starting point is to consider the equation of motion for the density autocorrelation function *Φ*_s_(*q*,*t*) = 〈*n*(−*q*,0)*n*(*q*,*t*)〉 of a selected particle at wave vector *q*,^44^31
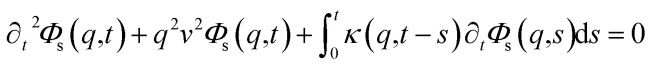
where 
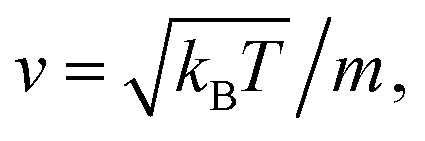
*k*_B_ stands for the Boltzmann constant, *T* denotes the temperature and *m* is the microemulsion mass.

The equation of motion for the MSD can be obtained from32
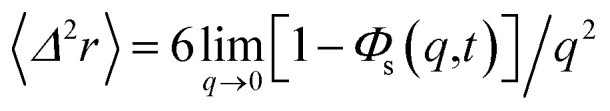
which gives33
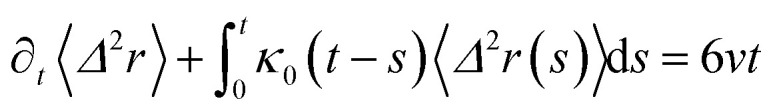
where 
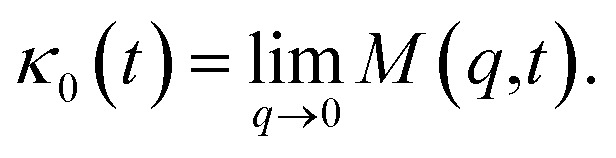


Thus, Flenner *et al.* propose a memory function with explicit crossover time scales which reproduce the three diffusion regimes, namely, ballistic, normal, and subdiffusive^44^34
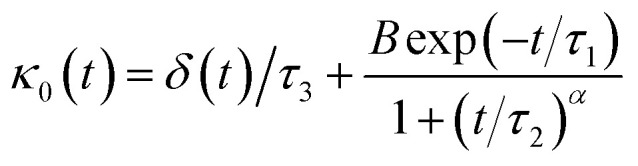
where *δ*(*t*) is the Dirac delta function, *B* is a dimensionless parameter, *τ*_1_ is the characteristic time for the crossover from subdiffusion to normal diffusion, *τ*_2_ is the onset time of the subdiffusion regime, and *τ*_3_ is the characteristic time for the crossover from the ballistic regime to the subdiffusive regime.

Analysis of this memory function revealed that, for *B* = 0, the Brownian diffusion laws are recovered and 〈*Δ*^2^*r*〉 behaves as35
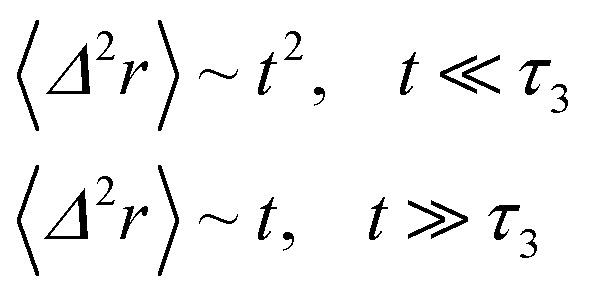
If (*t*/*τ*_2_)^*α*^ ≪ 136
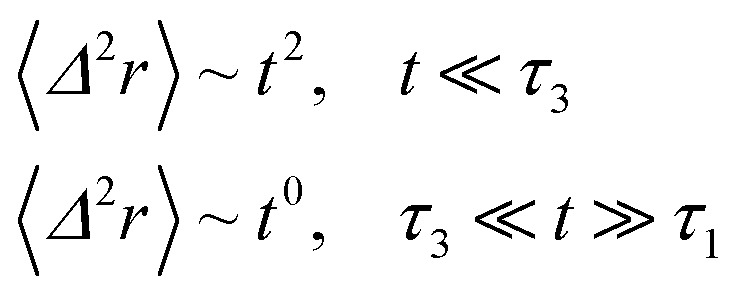


The subdiffusion phenomenon is represented mathematically in this memory function by the power law. The analytical determination of the diffusion exponent *α* seems to be complex. On the other hand, a numerical computation, taking into account the power law,^44^ revealed that the dynamics pass slowly from the ballistic regime to the subdiffusive regime with *α* < 1. The region of subdiffusion separating the ballistic and the normal regimes is due to a cage effect. In this region the mean-square displacement, 〈*Δ*^2^*r*〉, behaves as

37 〈*Δ*^2^*r*〉 ∼ *t*^*α*^ (*α* < 1), *t* > *τ*_2_

#### 3.3.3 MD simulation results

The influence of the volume fraction on the dynamic properties of decane/water microemulsions is investigated using NVT molecular dynamics simulations. For this study, the microemulsion radius, the effective charge of the microemulsion, and the temperature are fixed to the values: *R* = 62, *Z*_eff_ = 130, and T* = 1. But, the volume fraction varies over a large range.

**Fig. 7:**
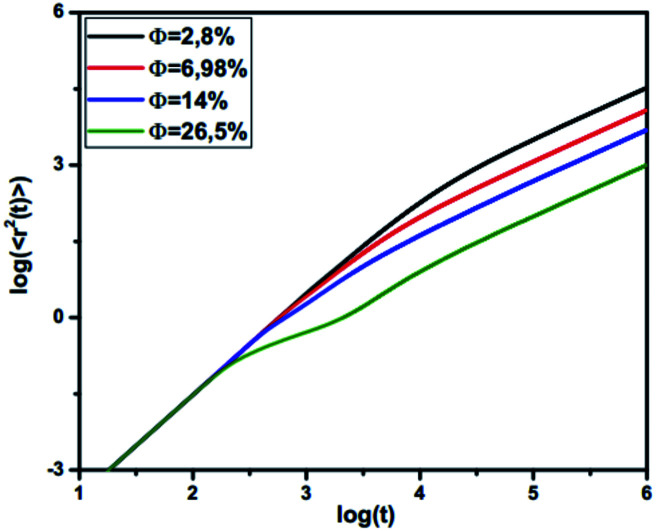
Log–log plot of MSD *versus* time step from MD simulation.

Fig. 7 shows the reduced MSD, Δ*r*_CM_^2^(*t*), as a function of reduced time, 
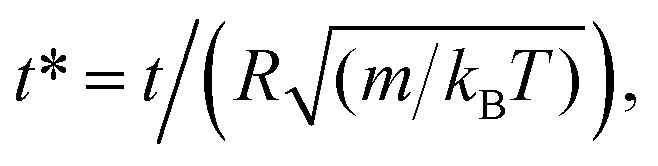
 on a log–log scale, for many values of volume fraction. For early times, we note that the curves are superimposable and the random-walker exhibits ballistic diffusion, and we have Δ*r*_CM_^2^(*t*) ∝ *t*^2^. Thus, the microemulsion diffusion is practically independent of the volume fraction. For later times, we note that the MSD depends on *Φ*. Accordingly, for low and medium volume fractions (*Φ* = 2.8%, 6.98%, 14%, 26.5%), for a reduced time below the transition time (τ*), the microemulsions experience ballistic diffusion. But after this transition time, the microemulsions exhibit normal diffusion. The obtained results are in good agreement with the theoretical predictions discussed previously (eqn (26) and (27)). This can be explained by the fact that at early times, the random-walker, referring to an individual microemulsion, does not feel the presence of its close neighbors yet. This diffusion regime is well known as Fickian diffusion.^32,45^

For high density microemulsions, ballistic diffusion is also observed for early times. However, for intermediate times beyond 
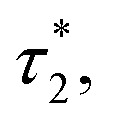
 the tracer, representing the considered microemulsion, is trapped in a cage formed by its close neighbors and then moves slowly. The associated MSD then behaves as Δ*r*_CM_^2^(*t*) ∝ *t*^*α*^, with subdiffusion exponent *α* = 0.47. This result conforms to the theoretical predictions we described above (eqn (37)). If the number of surrounding neighbors in the cage where the tracer is trapped is denoted by *N*_c_, the stay duration in this cage, Δ*t*_c_, scales as 
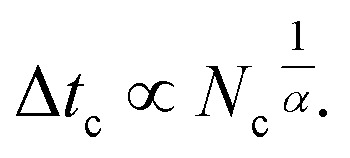
 Therefore, the stay duration in the cage becomes longer as the volume fraction is increased, because of the attraction that favors contact between the microemulsions and then blocks their movement. For high volume fractions, the available space for the microemulsions becomes smaller because of the strong attraction. This attraction then leads to slowing-down of the microemulsion scattering, which is localized for a certain time (stay duration in the cage). The latter increases with the volume fraction. After the subdiffusive regime, for 
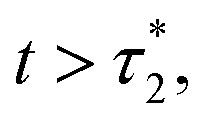
 the long-time diffusive regime (where Δ*r*_CM_^2^(*t*) ∝ *t*^*α*^, *α* = 1) is established. Generally, the ballistic and subdiffusive regimes do not last for a long time and a crossover to normal diffusion is always observed.^46,47^

**Fig. 8:**
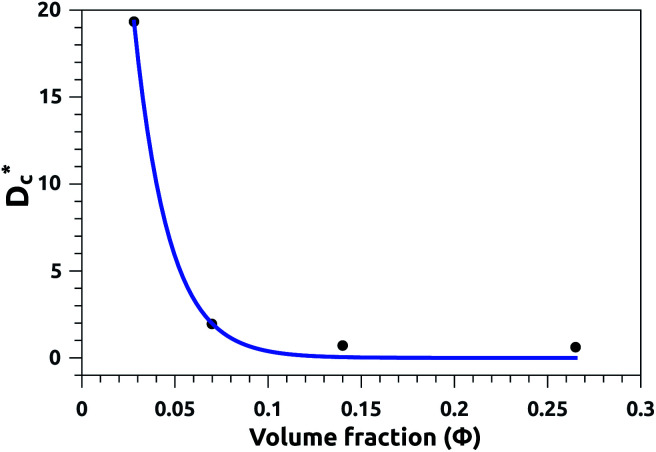
Diffusion coefficient 
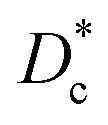
 as a function of the volume fraction *Φ*.

In Table 3, we give the normal diffusion coefficient, 
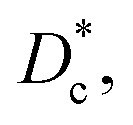
 for the studied microemulsions as a function of the volume fraction. We observe that, when *Φ* increases, *D*_c_ decreases exponentially. This can be explained by the fact that as *Φ* increases, the correlations between the microemulsions become larger. Similar dynamic behavior has been recently reported in MD simulations of a large number of colloidal particles.^48–52^ According to Fig. 8, 
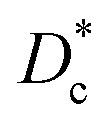
 decreases following an Arrhenius-like law, where:38
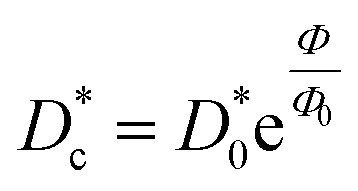
with 
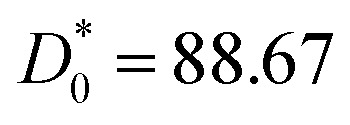
 and *Φ*_0_ = 0.0156. This behavior was observed in a recent MD simulation of Pickering microemulsions.^13^

**Table 3:** Dimensionless dynamic properties for different microemulsion volume fractions

*Φ*	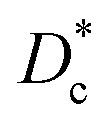	η*
2.8%	19.340	0.274 × 10^−2^
6.98%	1.952	2.718 × 10^−2^
14%	0.710	6.549 × 10^−2^
26.5%	0.615	32.15 × 10^−2^

## 4 Conclusion

In this work, we investigated the static and dynamic properties of positively charged spherical decane/water microemulsions using SANS experiments, the HNC numerical method, and NVT molecular dynamics simulations, for microemulsions with different volume fractions *Φ*. The calculated properties from the different approaches are found to be in good agreement, which confirms the appropriate choice and the correct parameterization of the interaction potential. It is noted that the static and dynamic properties of microemulsions strongly depend on *Φ*. On the one hand, the interaction between the microemulsions increases proportionally to *Φ*. On the other hand, the average distance between the microemulsions, the isothermal compressibility, and the diffusion coefficient decrease with increasing *Φ*.

It should be noted that the determination and parameterization of an effective interaction potential for studying colloidal particles is a real challenge. Although the DLVO model is complete, the parameterization of this potential model requires, at least, an experimental adjustment in terms of structural properties. Thus, the present procedure used to parameterize the interaction potential between the decane/water microemulsions stabilized by the CpCl surfactant and the octanol cosurfactant can be carried out for different types of colloidal particles. Then, molecular dynamics simulations can be used to determine several physical properties based on a proven effective interaction.

Finally, we propose that the present study can be extended to study microemulsions with biocompatible graft polymers on their surfaces, assuming that the incorporation of polymers can increase their stability and their diffusivity and thus increase the efficiency of microemulsions as drug carriers. This hypothesis will be the subject of our next investigations.

## Conflicts of interest

There are no conflicts to declare.

## Supplementary Material
